# Protocol for a single-arm, pilot trial of creatine monohydrate supplementation in patients with Alzheimer’s disease

**DOI:** 10.1186/s40814-024-01469-5

**Published:** 2024-02-27

**Authors:** Matthew K. Taylor, Jeffrey M. Burns, In-Young Choi, Trent J. Herda, Phil Lee, Aaron N. Smith, Debra K. Sullivan, Russell H. Swerdlow, Heather M. Wilkins

**Affiliations:** 1 Department of Dietetics and Nutrition, University of Kansas Medical Center, Kansas City, KS 66160 USA; 2 Alzheimer’s Disease Research Center, University of Kansas, Fairway, KS 66205 USA; 3 Department of Neurology, University of Kansas Medical Center, Kansas City, KS 66160 USA; 4 Hoglund Biomedical Imaging Center, University of Kansas Medical Center, Kansas City, KS 66160 USA; 5 Department of Radiology, University of Kansas Medical Center, Kansas City, KS 66160 USA; 6 Department of Health, Sport, and Exercise Sciences, University of Kansas, Lawrence, KS 66045 USA; 7 Department of Cell Biology and Physiology, University of Kansas Medical Center, Kansas City, KS 66160 USA; 8 Department of Biochemistry and Molecular Biology, University of Kansas Medical Center, Kansas City, KS 66160 USA

**Keywords:** Alzheimer’s disease, Creatine, Brain, Bioenergetics, Pilot trial

## Abstract

**Background:**

Impaired brain bioenergetics is a pathological hallmark of Alzheimer’s disease (AD) and is a compelling target for AD treatment. Patients with AD exhibit dysfunction in the brain creatine (Cr) system, which is integral in maintaining bioenergetic flux. Recent studies in AD mouse models suggest Cr supplementation improves brain mitochondrial function and may be protective of AD peptide pathology and cognition.

**Aims:**

The *Creatine to Augment Bioenergetics in Alzheimer’s disease (CABA)* study is designed to primarily assess the feasibility of supplementation with 20 g/day of creatine monohydrate (CrM) in patients with cognitive impairment due to AD. Secondary aims are designed to generate preliminary data investigating changes in brain Cr levels, cognition, peripheral and brain mitochondrial function, and muscle strength and size.

**Methods:**

CABA is an 8-week, single-arm pilot study that will recruit 20 patients with cognitive impairment due to AD. Participants attend five in-person study visits: two visits at baseline to conduct screening and baseline assessments, a 4-week visit, and two 8-week visits. Outcomes assessment includes recruitment, retention, and compliance, cognitive testing, magnetic resonance spectroscopy of brain metabolites, platelet and lymphocyte mitochondrial function, and muscle strength and morphology at baseline and 8 weeks.

**Discussion:**

CABA is the first study to investigate CrM as a potential treatment in patients with AD. The pilot data generated by this study are pertinent to inform the design of future large-scale efficacy trials.

**Trial registration:**

ClinicalTrials.gov, NCT05383833, registered on 20 May 2022.

## Introduction

Alzheimer’s disease (AD) affects more than 1 in 8 Americans over the age of 65 and is expected to grow in prevalence over the coming decades [1]. Historically, the pipeline of therapy development has been relatively futile, leaving few therapeutic options for patients with AD [2]. Much effort in advancing therapies in AD has been focused on halting deposition of and removing amyloid-beta (Aβ) plaque deposits in the brain, a pathological hallmark in the development of AD. Although recent trials suggest new treatments may be promising for removing Aβ from the brain [3, 4] and modestly improving cognitive symptoms in humans with AD [5], it is likely that additional therapies will also be required to treat and prevent symptomatic AD.

Interventions that target brain energy metabolism are also emerging as promising disease modifying therapy approaches for AD and for its prevention [6, 7]. Impaired brain bioenergetics precede symptomatic AD and are increasingly acknowledged as a contributor to its pathology [8]. Patients with AD exhibit dysfunction in the brain creatine (Cr) system [9], an integral system for maintaining brain energy flux. In the brain, free Cr is phosphorylated to form phosphocreatine (PCr), a primary means of storing and transporting high-energy phosphates derived from both cytosolic and mitochondrial metabolism [10]. In AD, the creatine kinase brain isoenzyme (BB-CK) responsible for phosphorylating Cr in the cytosol is severely reduced [11], as are both free Cr and PCr [12]. Impairments in the brain Cr system in AD may limit the ability to meet the high constant energy demand of the brain and potentially signal mitochondria to downregulate ATP production [13].

Creatine monohydrate (CrM) is an oral nutritional supplement that has been used extensively as an ergogenic aid for sport and exercise and has had growing interest in its possible brain health benefits [14]. It safely increases Cr levels in skeletal muscle [15], yet its effect on brain Cr levels has been inconsistent in the few small studies that have been completed with varying doses and durations [16–21]. In humans, limited but encouraging data suggest that CrM improves cognition in both younger and older adults [22], particularly in pathological brain conditions [23]. In animals, CrM supplementation has been shown to preserve bioenergetic function of hippocampal neurons and protect against brain aggregation of Aβ [24]. Recently, an 8-week CrM intervention in the 3xTg mouse model of AD improved hippocampal mitochondrial respiration in both male and female mice [25]. Female mice also improved cognition and decreased Aβ peptide aggregation in the hippocampus [25]. These data suggest that CrM improves the symptoms associated with AD in a mouse model.

Despite evidence in animal models that CrM may be neuroprotective, there have been no trials investigating CrM as a potential treatment in patients with AD. We are currently conducting a single-arm open-label pilot trial, *Creatine to Augment Bioenergetics in Alzheimer’s disease* (CABA), designed to investigate the feasibility of CrM supplementation and generate preliminary efficacy data pertaining to both function and mechanisms in the brain and periphery in patients with AD.

## Study aims and objectives

The primary aim of CABA is to investigate whether 8 weeks of 20 g/day of CrM supplementation is feasible in patients with dementia due to AD. Our secondary aims are to generate preliminary data investigating changes in (1) total creatine concentration in the brain, (2) cognition, (3) mitochondrial biomarkers in the periphery and brain, and (4) muscle function and morphology coincident with the 8-week CrM intervention.

## Design and method

The CABA protocol follows the guidelines for reporting non-randomized pilot and feasibility trials [26]. The study protocol has been approved by the Institutional Review Board at the University of Kansas Medical Center, and the trial has been registered with ClinicalTrials.gov (NCT05383833).

### Trial design

CABA is a single-arm, open-label, pre-post pilot trial. We will recruit 20 participants with AD, and all will be assigned to the 20 g/day CrM supplement intervention for 8 weeks. This dosage has been shown to be feasible and safe in healthy older adults, and limited data suggest that it may raise Cr levels in the brain [22].

### Trial setting

CABA is a single-center trial at the University of Kansas Medical Center (KUMC) in Kansas City, KS. All study visits occur in person at the Hoglund Biomedical Imaging Center and the Landon Center on Aging, on campus buildings adjacent to each other in close proximity.

### Sample size

The primary aim of CABA is to investigate the feasibility of the CrM intervention; thus, formal estimates of power are not required [27]. This is the first human trial to investigate CrM supplementation in patients with AD. The secondary aims of CABA are designed to generate means and confidence estimates that inform sample size requirements of subsequent studies. With a sample size of 20, we will be able to estimate an 80% compliance rate with a 95% confidence interval of 62.5–97.5% and establish effect estimates and confidence intervals that can be used to inform the design of future large clinical trials.

### Participant eligibility criteria

The CABA trial aims to enroll an equal sample of males and females that have been diagnosed with cognitive impairment due to AD [28] with a mini-mental state exam (MMSE) score ≥ 17. Prior to enrollment, participants require stable dosage of AD-related medications (i.e., donepezil, memantine) for 30 days. Participants also require the cooperation of a study partner (spouse, relative, or close friend) to help facilitate the intervention and accompany the participant to all study visits. Table 1 summarizes the trial inclusion and exclusion criteria.

**Table 1:** Participant eligibility criteria

**Inclusion criteria**
• Diagnosed cognitive impairment due to AD [28] • Agreed cooperation from a study partner • Speaks English as primary language • Age 60 to 90 • Stable AD medication for ≥ 30 days • BMI ≥ 18.5 kg/m^2^
**Exclusion criteria**
• Diabetes (types 1 or 2), cancer requiring chemotherapy or radiation within the past 5 years, or recent cardiac event (i.e., heart attack) • Other neurodegenerative disease • Ongoing renal disorder or abnormal renal or liver function • Use of medications or supplements that affect blood glucose (i.e., metformin) • Unable to undergo MRI • Clinical trial or investigational drug or therapy participation within 30 days of the screening visit • Non-English speakers • Inability to perform strength testing • Weight > 350 lbs • MMSE < 17

### Recruitment

**Fig. 1:**
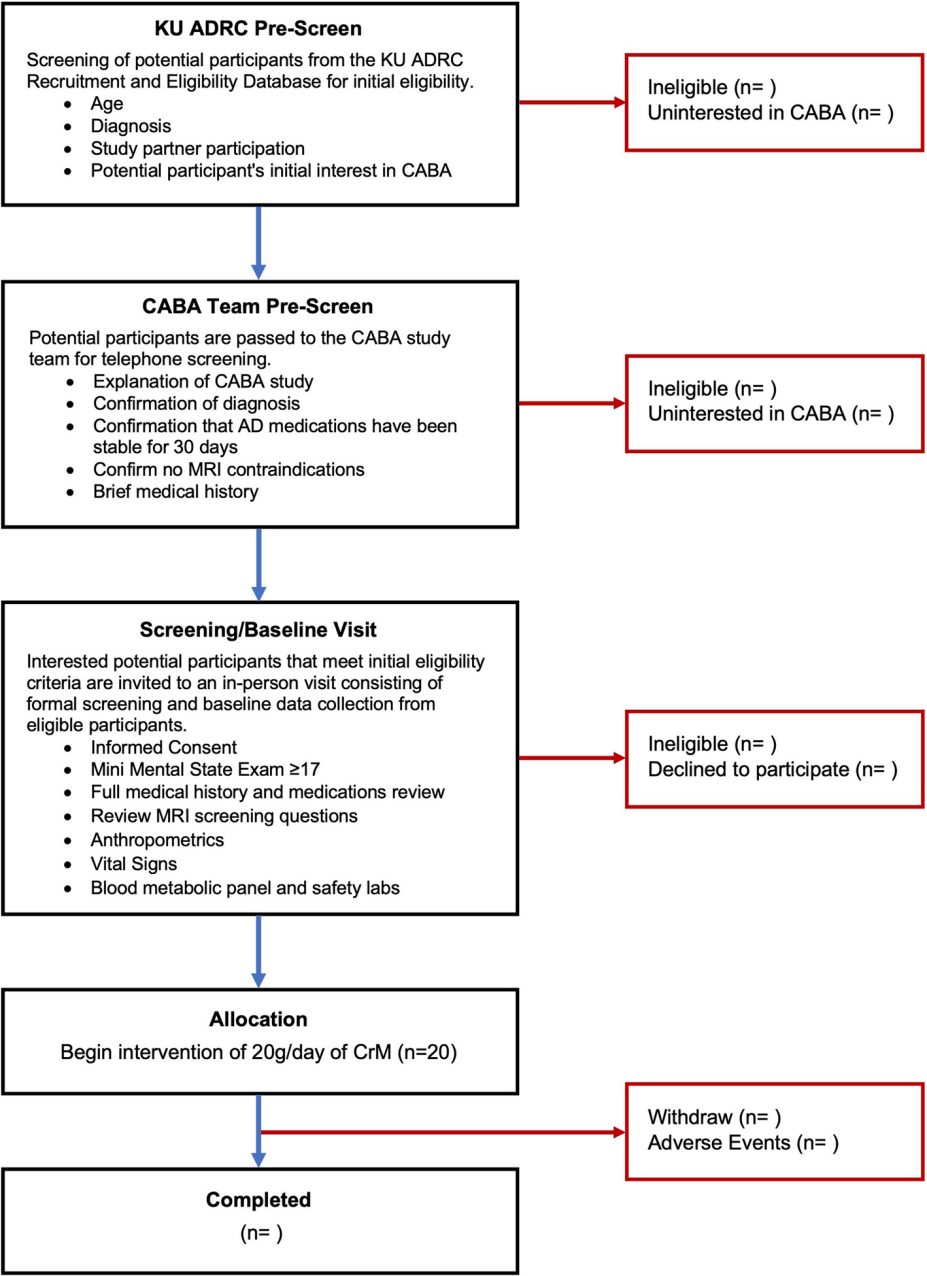
CABA recruitment and screening flow

Recruitment for CABA leverages the existing recruitment infrastructure at the KU Alzheimer’s Disease Research Center (KU ADRC) [29]. The KU ADRC Outreach, Recruitment, and Engagement (ORE) Core initially pre-screens potential participants from a database of individuals that have consented to be contacted by the KU ADRC about study opportunities. Potential participants that meet preliminary diagnostic criteria are then phone screened by the CABA study team to explain the study and attain brief medical history. Individuals that are interested in participating in CABA and meet initial eligibility requirements are invited to attend the formal screening/baseline visit to attain informed consent and confirm eligibility. The CABA recruitment flow is illustrated in Fig. 1.

### Intervention

Delivery of the intervention is facilitated by registered dietitians (RD). After all baseline assessments have been completed, RDs provide participants and their study partners with a 5-week supply of CrM powder (Life Extension, USA) along with written and verbal instruction on how to consume the CrM. Participants are instructed to stir 10 g (1 scoop) of CrM powder into liquid to consume in the midmorning and to repeat the same process in the evening for a total of 20 g each day. All participants receive another 5-week supply of CrM at the 4-week study visit, which provides enough CrM to each participant to complete the study. The additional week supply of CrM is intended to cover any error in study visit scheduling by 1 week (i.e., the 4-week visit at week 5 of intervention). Study partners are asked to assist participants in completion of a daily supplement calendar designed to track consumption of the two daily doses of CrM for the duration of the study. To encourage compliance and monitor for adverse events (AEs), the study RDs speak with participants and their study partners each week during planned telephone calls.

### Study visits and assessment timeline

Study flow and a description of the study visits and procedures are presented in Table 2. All study procedures take place over five visits: one screening/baseline visit, a second baseline visit within 1 to 2 weeks of the first visit, a 4-week visit, and two 8-week visits that occur within 1 week of each other.

**Table 2:**
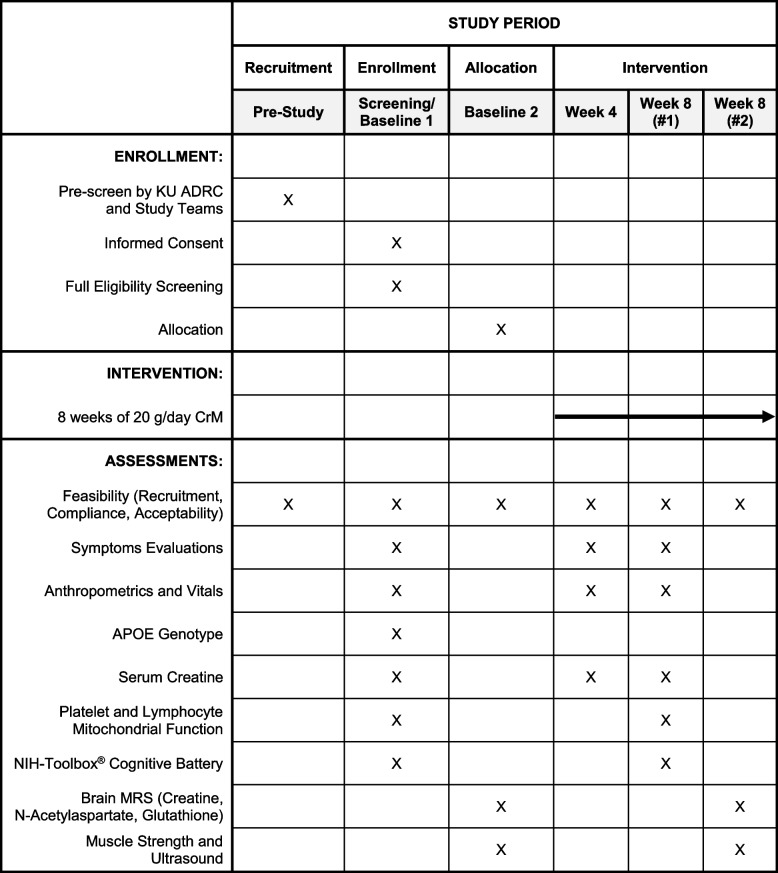
Study visit procedure summary

## Assessments

All study assessments and their associated objectives are presented in Table 3.

**Table 3:** Study objectives and outcomes

Objective	Measurement	Timepoint
Aim 1: Feasibility	Recruitment and retention	Throughout study
	Self-reported compliance on Cr supplement calendar	Throughout intervention
	Serum creatine (Cr) levels	Baseline, 4 weeks, and 8 weeks
Aim 2: Brain creatine concentration	Magnetic resonance spectroscopy measurement of brain total creatine (Cr)	Baseline and 8 weeks
Aim 3: Cognition	NIH-Toolbox® Cognitive Battery	Baseline and 8 weeks
Aim 4: Peripheral mitochondrial function	OROBOROS Oxygraph-2 K measurement of platelet and lymphocyte respiration, ATP production, and ROS production	Baseline and 8 weeks
Aim 4: Brain mitochondrial function	Magnetic resonance spectroscopy measurement of brain N-acetylaspartate (NAA) and glutathione (GSH)	Baseline and 8 weeks
Aim 5: Muscle strength and morphology	Handgrip strength, leg extensor strength, and quadriceps ultrasound	Baseline and 8 weeks

### Feasibility

The feasibility of the intervention will be based on compliance and safety. We also track the number of participants who are pre-screened for preliminary eligibility, contacted for recruitment, consent to enroll in the study, and withdraw from the study before or after allocation (Fig. 1).

The primary compliance measure will be based on participant completion of the study along with daily reported CrM intake on provided supplement calendars. Our criterion for compliance feasibility is constituted as ≥ 80% compliance with daily supplement intake among ≥ 80% of the study sample (*n* ≥ 16). Participants also bring the remainder of unconsumed CrM to 4-week and 8-week visits to be weighed by the study team to aid in determining compliance.

### Safety

Safety is assessed by frequency and severity of reported AEs, proportion of participant withdrawals due to AEs, and monitoring of blood safety labs. Study personnel ascertain AEs and side effects during weekly telephone calls and at the 4-week and 8-week study visits. Participants also have continuous access to study personnel, which allows them to report potential AEs on a continuous basis. All AEs are immediately reported to the PI; assessed for severity, cause, and expectedness; and followed until resolution. Additional safety checks include monitoring change in lab values from a comprehensive metabolic panel and liver and renal function tests (Quest Diagnostics, USA).

### Cognitive testing

The mini-mental state exam (MMSE), a brief, multidomain cognitive test and dementia screening tool, is administered by trained study personnel at baseline as part of the screening procedures and again at 8 weeks.

Comprehensive cognitive testing is also completed at baseline and 8 weeks using the NIH Toolbox® (NIH-TB) cognitive battery [30]. NIH-TB has been validated in both cognitively normal and demented older adults [31]. The cognitive battery is facilitated by trained staff on an iPad (Apple, USA) and contains individual tests from cognitive domains of attention, category switching, episodic memory, working memory, speed of processing, written language, and auditory language. Unadjusted, age‐adjusted, and fully adjusted (age, sex, education) scores are automatically calculated for each individual test as well as a composite cognition score.

### Magnetic resonance imaging

High-resolution T1-weighted MRI is acquired using three-dimensional (3D) magnetization prepared rapid acquisition with gradient echo (MPRAGE) sequence (sagittal, 1 mm isotropic resolution, *TE/TI/TR* = 3.98/830/2000 ms, *FOV* = 256 × 256 mm^2^, 176 slices). The purpose of our T1-weighted MRI is to co-register magnetic resonance spectroscopy (MRS) scans and correct partial volume effects in MRS quantification.

### Magnetic resonance spectroscopy

Brain total Cr (tCr), N-acetylaspartate (NAA), and glutathione (GSH) concentrations are measured via MRS at baseline and 8 weeks on a 3 T MR system (Skyra, Siemens, Erlangen, Germany) using a 20-channel receiver RF coil, selecting an axial slab of the frontal and parietal regions above the lateral ventricle. Measuring brain tCr will provide insight into the effectiveness of our CrM intervention to increase brain Cr concentration. GSH is an important antioxidant defense system that reflects oxidative stress status [32] and, together with NAA, will provide insight into brain mitochondrial integrity [33, 34]. Our semi-LASER MRSI sequence for Cr and NAA (*TE/TR* = 38/2000 ms, *FOV* = 20 cm, matrix = 14 × 14, slice thickness = 25 mm, *VOI* = 80 × 80 mm^2^) and doubly selective multiple quantum-filtered MRSI sequence for GSH (TE/TR = 115/1750 ms, *FOV* = 200 mm, matrix = 10 × 10, slice thickness = 25 mm) [35, 36] minimize the effect of MR system instability and subject motion during the scan [37]. Metabolite signals from the frontal, parietal, and frontoparietal regions are quantified with LCModel software using water signals from the same slab of the brain as an internal frequency and concentration reference [38]. Metabolite concentrations in the gray and white matter are also calculated using an established regression method [39].

### Blood measures

Blood is drawn after an 8-h fast at baseline, 4-week, and 8-week study visits to perform several different assays.

At baseline, apolipoprotein E (APOE) genotype is determined using a TaqMan single-nucleotide polymorphism (SNP) allelic discrimination assay (ThermoFisher). APOE2, APOE3, and APOE4 alleles are determined using TaqMan probes at the two APOE-defining SNPs, rs429358 (C_3084793_20) and rs7412 (C_904973_10).

At baseline, 4 weeks, and 8 weeks, serum creatine is quantified via enzymatic assay (Quest Diagnostics). We expect serum creatine levels to increase as a result of the intervention, which can be used as an objective biomarker of compliance.

A comprehensive metabolic panel including liver and renal function tests (Quest Diagnostics) is measured at baseline and 8 weeks to monitor safety of the intervention.

Blood-based mitochondrial biomarkers are measured by the KU ADRC Biomarker Core at baseline and 8 weeks. Blood is collected into acid citrate dextrose tubes and fresh platelets, and lymphocytes are promptly isolated from the samples. Platelet and lymphocyte plasma membranes are permeabilized by adding digitonin, and the OROBOROS Oxygraph-2 k (OROBOROS Instruments, Austria) instrument is used to measure platelet and lymphocyte mitochondrial oxygen respiration kinetics. Reactive oxygen species (ROS) is measured using Amplex Red and MitoSOX with flow cytometry. ATP and ADP levels are measured using a bioluminescent assay.

### Muscle function and morphology

Muscle function is assessed at baseline and 8 weeks using handgrip strength and leg extensor strength testing. Handgrip strength is measured as the average of three consecutive tests with a hand dynamometer (Lafayette Instrument, USA) while seated in a chair with feet resting on the floor. The study team verbally encourages participants to squeeze the dynamometer with maximal effort for at least 3 s. Leg extensor strength is measured by completing five maximal isokinetic contractions of the right leg extensors at three different velocities (1.05 rad·s^−1^, 2.09 rad·s^−1^, and 3.14 rad·s^−1^) on a calibrated isokinetic dynamometer (Biodex Medical Systems, Inc., USA) [40]. Study personnel verbally encourage participants to work as hard and as fast as possible during each repetition. Torque, position, and velocity signals from leg extensor strength testing are recorded.

Muscle morphology is assessed at baseline and 8 weeks using ultrasonography (GE Healthcare UK, UK) to measure muscle cross-sectional area (mCSA) and echo intensity (mEI) of the right quadriceps muscles, while participants lie supine. All ultrasound imaging analyses are performed using ImageJ software (Version 1.46r, National Institutes of Health, Bethesda, MD, USA).

### Progression to a larger clinical trial

Meeting our primary feasibility goal of 80% compliance with the intervention protocol (*n* ≥ 16 participants that report consuming ≥ 80% of the CrM doses) will constitute a successful study, regarding feasibility. To support progression to a future randomized, controlled trial investigating differential effects of CrM and placebo on brain health outcomes in patients with AD will require both demonstration of feasibility and a positive signal from any of our secondary physiology or cognition outcomes. These measures were designed to generate preliminary data on symptoms and physiology that may be relevant to AD; thus, any changes associated with the CrM intervention would warrant broader, large-scale investigation.

## Data analysis

Demographic characteristics of study participants will be presented as means and standard deviations for continuous variables and frequencies and proportions for categorical variables.

The aim of this study is to determine feasibility of the CrM intervention and outcomes assessments in patients with AD. Our primary outcome will be compliance, which we consider compliance with the intervention among 80% of the study sample to be satisfactory. We will also use our recruitment tracking pipeline to determine the length of time required to enroll our full sample, the proportion of interested participants that consented to enroll in the study, and the reasons for non-recruitment, non-enrollment, and non-compliance.

We will also explore preliminary data regarding change in several AD-related secondary outcomes. Since all participants receive the CrM intervention, we will conduct paired samples *t*-tests for all outcomes to estimate means and 95% confidence intervals of within-group change from baseline. If model assumptions are not met, we will alternatively use the nonparametric paired Wilcoxon test to report medians and interquartile ranges. Statistical analyses will be performed using R (v. 4.2.1; R Foundation, Vienna, Austria).

## Data management and security

Data is collected both on paper and electronically. All paper data are stored securely in a locked filing cabinet at KUMC. All electronic data is managed using the Research Electronic Data Capture (REDCap) [41, 42] system hosted by a HIPAA-complaint server. Data is entered by the research staff and validated with real-time entry validation and offline validation by the investigators. The study team is assigned role-based access to REDCap which is protected by institutional multi-factor password protection.

All data is gathered for the explicit purposes of this study using procedures to ensure confidentiality. Within the REDCap database, participant identifiers are available to the study interventionalists only as they will be responsible for participant scheduling and delivery of the intervention. For all other study investigators, all data is de-identified and organized by participants’ assigned study IDs only. All data for publication or presentation will also be deidentified.

## Dissemination

The results of this study will be published in peer-reviewed scientific journals and will be presented at international conferences, such as the Alzheimer’s Association’s International Conference. The research team will agree upon authorship in advance of manuscript submission. We will include statistical code used for data analyses as supplemental data with submitted manuscripts. Final de-identified data will available upon request to the principal investigator, contingent upon completion of a data sharing agreement with the University of Kansas Medical Center.

## Discussion

To date, the CABA study is the first to investigate CrM supplementation in patients with AD. Investigating CrM as an adjuvant therapy in AD is supported by recent positive animal studies and may influence several bioenergetic-related targets including mitochondrial function, oxidative stress, and inflammation. This pilot trial will provide information regarding the feasibility of the CrM intervention and the study outcomes, collectively, to inform the optimal design of a future clinical trial.

The CABA trial is not designed to determine efficacy of the CrM intervention in AD; thus, any results from its secondary outcomes should be interpreted with caution. The single-arm design of this study does not allow for comparison of change in secondary outcomes with a control group. This study also features a relatively small sample size along with measurement of several physiologic variables, which increases the chance for type I error due to multiplicity. However, since AD is a progressive disease, without intervention, we would expect either decline or no change in cognition from our participants. We also would not expect brain tCr levels or mitochondrial function to increase substantially with no intervention. Therefore, within-group signal for improvement in our secondary outcomes would provide further rationale for testing with larger studies. CABA is an important first step in generating effect estimates and confidence intervals to aid in determining the appropriate sample size necessary for hypothesis testing in a subsequent clinical trial.

## Trial status

This manuscript reflects protocol version 2 dated March 17, 2023. Recruitment began in December 2022. We anticipate completion of all trial assessments by the end of June 2024.

## Data Availability

Not applicable. No datasets were generated or analyzed.

## Abbreviations

AD: Alzheimer’s disease
AE: Adverse event
APOE: Apolipoprotein E
ATP: Adenosine triphosphate
BB-CK: Creatine kinase brain isoenzyme
BMI: Body mass index
CABA: Creatine to Augment Bioenergetics in Alzheimer’s disease
CBF: Cerebral blood flow
Cr: Creatine
CrM: Creatine monohydrate
DHQ III: Diet History Questionnaire III
GSH: Glutathione
mCSA: Muscle cross-sectional area
mEI: Muscle echo intensity
MMSE: Mini-mental state exam
MRI: Magnetic resonance imaging
MRS: Magnetic resonance spectroscopy
NAA: N-Acetylaspartate
NIH-TB: NIH Toolbox®
PCr: Phosphocreatine
tCr: Total creatine
